# Targeted enrichment by solution-based hybrid capture to identify genetic sequence variants in barley

**DOI:** 10.1038/s41597-019-0011-z

**Published:** 2019-04-01

**Authors:** Camilla Beate Hill, Debbie Wong, Josquin Tibbits, Kerrie Forrest, Matthew Hayden, Xiao-Qi Zhang, Sharon Westcott, Tefera Tolera Angessa, Chengdao Li

**Affiliations:** 1 Western Barley Genetics Alliance, Western Australian State Agricultural Biotechnology Centre, School of Veterinary and Life Sciences, Murdoch University, Murdoch, WA Australia; 2 Agriculture Victoria, Agriculture Research Division, AgriBio, Centre for AgriBioscience, Bundoora, VIC Australia; 3 School of Applied Systems Biology, La Trobe University, Bundoora, VIC Australia; 4 Agriculture and Food, Department of Primary Industries and Regional Development, South Perth, WA Australia

**Keywords:** Agricultural genetics, Targeted resequencing, Next-generation sequencing, Genetic markers

## Abstract

In barley and other cereal crops, phenological diversity drives adaptation to different cultivation areas. Improvement of barley yield and quality traits requires adaptation to specific production areas with introgression of favorable alleles dependent upon precise identification of the underlying genes. Combining targeted sequence capture systems with next-generation sequencing provides an efficient approach to explore target genetic regions at high resolution, and allows rapid discovery of thousands of genetic polymorphisms. Here, we apply a versatile target-capture method to detect genome-wide polymorphisms in 174 flowering time-related genes, chosen based on prior knowledge from barley, rice, and *Arabidopsis thaliana*. Sequences were generated across a phenologically diverse panel of 895 barley varieties, resulting a high mean depth coverage of ~25x allowing reliable discovery and calling of insertion-deletion (InDel) and single nucleotide polymorphisms (SNPs). Sequences of InDel and SNPs from the targeted enrichment were utilized to develop 67 Kompetitive Allele Specific PCR (KASP) markers for validation. This work provides researchers and breeders a comprehensive molecular toolkit for the selection of phenology-related traits in barley.

## Abstract (summary)

**Table:** 

Design Type(s)	genetic polymorphism analysis objective • strain comparison design
Measurement Type(s)	genetic variation
Technology Type(s)	DNA sequencing
Factor Type(s)	cultivar
Sample Characteristic(s)	Hordeum vulgare subsp. vulgare • leaf

Machine-accessible metadata file describing the reported data (ISA-Tab format)

## Background & Summary

Phenological diversity drives adaptation to different areas of cultivation in barley and other cereal crops^1^. The triggering of flowering is influenced by endogenous genetic and exogenous environmental cues, including day length and temperature^2^. The timing of flowering directly influences grain yield, as it needs optimisation to occur during specific seasons to avoid environmental stresses such as frost, heat, and drought. Extensive research in the model plant *Arabidopsis thaliana* have revealed key genetic mechanisms that control flowering. Although many of the circadian clock genes are conserved between *Arabidopsis* and barley, there are many differences specific to cereal grasses^3^.

Improvement of barley yield and quality traits, enhancing adaptation to specific production areas, and introgression of favorable alleles into commercial varieties all depend upon the precise identification of the underlying genes^2^. Differences in many alleles that modify phenology are based on genetic variants relative to the barley reference genome. The recent availability of a barley genome assembly with high-confidence sequences^4^ allows the identification of phenology-associated gene orthologs.

Advances in second-generation sequencing technologies and bioinformatics techniques in the last decade now provide methodologies to interrogate genome diversity for many plant accessions. However, for plants with large and highly repetitive genomes including many cereal crops, technical challenges and economic barriers can prevent genome re-sequencing on a population scale to a sufficient depth. Reduced-representation sequencing approaches that access a focused subset of loci within a genome, including exome capture, RNA sequencing (RNA-seq), and target capture approaches, can be applied to any species for which a draft or complete reference genome sequence is available^5^. Exome capture approaches can identify sequence variants only in protein-coding genome regions, and RNA-seq can only be used to interrogate transcriptome diversity of genes expressed in the sampled tissue. Target capture based on in-solution hybridization allows for a cost-effective identification of sequence variants in coding- and noncoding regions of very large genomes. It involves custom design of capture probes targeting specific chromosome regions harbouring loci or candidate genes for traits of interest, which enable extremely flexible scaling of resequencing experiments of few to many genes at low cost for large plant populations.

The aim of this project is to provide gene sequences of ca. 174 flowering time regulatory genes and gene orthologs across a large barley population of 895 accessions for the purpose of identifying phenology gene sequence variations across current barley breeding germplasms. To achieve this, we used target capture based on in-solution hybridization, a method that focuses only a subset of the genome and thus significantly reduces the sequencing space and cost. The myBaits^®^ technology (Arbor Biosciences, Ann Arbour, MI, USA) which is based on in-solution biotinylated RNA probes was used for target enrichment. The resulting sequence capture libraries were subjected to Illumina sequencing in paired-end mode. Baits were designed for flowering time regulatory genes and gene homologs, and used for production of 80mer capture oligonucleotides for hybridization. Sequencing was performed for 895 diverse barley accessions selected from a large collection of landrace, cultivated barley, and research varieties of diverse origin. This set represents the whole variety of cultivated barley lifeforms, namely two- and six-row genotypes with winter, spring, and facultative growth habits. The results show abundant genetic variation in the form of SNPs and InDels in the different barley varieties. Sequences of InDels and SNPs from the targeted enrichment were utilized to develop 67 Kompetitive Allele Specific PCR (KASP) markers to detect and distinguish cultivar-specific alleles for candidate phenology genes in a subset of the re-sequenced barley varieties.

The ability to fine-tune flowering to the growing season could give significant advantages for both breeders and growers. Marker-assisted selection (MAS) and targeted genetic modification of flowering behaviour combined with faster breeding cycles have great potential to optimise the timing of flowering to specific environments and maximise grain yield^6^. The availability of this dataset will serve as a valuable resource to the cereal research community, and will help identify valuable traits to assist in breeding programmes.

An accompanying publication describes the biological implications of the data^7^. We have deposited the relevant raw data to public sequence data archives in Array Express^8^.

## Methods

### Plant material

As described in ref.^7^, the barley accessions of the phenology diversity panel of core barley varieties were selected from a large collection of over 4,000 landrace, cultivated, and research barley varieties of diverse origin preserved at the Western Barley Genetics Alliance at Murdoch University (Perth, Australia). The selection of barley accessions included domesticated, landrace, and breeding accessions from 41 countries, and was based on diversity in phenology development, and diverse geographic origins to represent European, Asian, North and South American, and Australian breeding programs (Germplasm.xlsx^9^). The selection of domesticated barley originated from various breeding programs and represented the whole variety of cultivated barley lifeforms, including two- and six-row genotypes with winter, spring, and facultative growth habits.

### Design of the targeted enrichment assay

We designed a custom target enrichment sequencing assay that included the loci implicated in the flowering pathways in barley and related plant species (described in ref.^7^). A set of genic sequences comprised a comprehensive subset of loci related to flowering time and development of meristem and inflorescences, including circadian clock regulators (*TIMING OF CAB EXPRESSION 1* (*TOC1*), *CYCLING DOF FACTOR 1* (*CDF1*), *EARLY FLOWERING 3* (*ELF3*), *GIGANTEA* (*GI*), and *ZEITLUPE a* and *ZEITLUPE b* (*ZTLa/b*)), the input pathways for vernalisation (*EARLY FLOWERING 7* (*ELF7*), *EARLY FLOWERING IN SHORT DAYS* (*EFS*), *VERNALISATION H1* (*VRN-H1*), *VERNALISATION INSENSITIVE 3* (*VIN3*)), photoperiod sensitivity (*CONSTANS 1* (*CO1*), *CRYPTOCHROMES 1a*, *1b*, *2* (*CRY1a/b*, *CRY2*), *PHYTOCHROMES A,B*, and *C* (*PHYA*, *PHYB*, *PHYC*)) and gibberellin (*GIBBERELLIN-20-OXIDASE 2* (*GA20ox2*), *GIBBERELLIN-2-OXIDASE 3* (*GA2ox3*)), along with downstream signal transducers (*APETALA 2* (*AP2*), *FLOWERING LOCUS D* (FD), *FLOWERING LOCUS T* (*FT*), *LEAFY 1* (*LFY1*), *SQUAMOSA PROMOTOR PROTEIN LIKE 3* (*SPL3*), *SUPPRESSOR OF CONSTANS 1* (*SOC1*), *TEMPRANILLO 1* (*TEM1*), *TERMINAL FLOWER 1* (*TFL1*)). Additionally, it contained a selection of genes related to grain yield and quality traits including *SERINE/THREONINE PROTEIN PHOSPHATASE GENE* (*PPP*), *WRKY DNA-BINDING PROTEIN* (*WRKY61*), and *TUBULIN ALPHA-3 CHAIN* (*TUBA3*) (Phenology_Genes.xlsx^9^). Scientific literature was mined for the genes implicated in flowering time pathways in barley and the corresponding nucleotide sequences were extracted from NCBI GenBank. In addition, barley homologs of flowering genes from the other grass species, such as bread wheat and rice, were identified through BLASTN search (e-value < 1e^−10^) and included. Complete genomic DNA (gDNA) sequences were selected for probe design. If the complete gDNA was absent, partial cDNA and cDNA sequences were included instead.

### Probe design

Targeted enrichment of genomic DNA regions was performed by solution-based hybrid capture using a synthetic library consisting of a final set of 13,588 probes (myBaits®, Arbor Biosciences, Ann Arbour, MI, USA; http://www.arborbiosci.com) following the manufacturer’s protocol version 3.01 (http://www.mycroarray.com/pdf/MYbaits-manual-v3.pdf; accessed 14/02/2018). As described in ref.^7^, the capture oligonucleotides were 80 nt long and were designed to target approximately 600 kb of repeat- and organelle-masked genetic sequence (190 phenology-related genes, derived from *Hordeum vulgare* L. genome assembly 082214v1) in with a 2x tiling density. Probes were designed from the FASTA sequences of each gene. For probe design, the sequence of the gene was repeat-masked based on organelle, transposable elements^10^ (TREP), and high copy number in the barley reference genome (genome assembly 082214v1). Probes based on intron-flanking exons were designed as they would still capture fragments covering the entire intron for genes with small introns (<500 bp) if probes based on intron sequence failed to capture fragments.

### Library Preparation and target enrichment

Genomic DNA was extracted from leaves of a single two-week old barley plant per variety using the cetyl-trimethyl-ammonium bromide (CTAB) method^11^. DNA concentration was determined using a nanodrop spectrophotometer (Thermo Scientific) according to the manufacturer’s protocol. DNA quantity and purity was further checked on 0.5% agarose gel (100 V, 0.5 × TBE, 60 min). Physical shearing of genomic DNA was performed with an average size of 275 bp using a Covaris S2 Focused-Ultrasonicator. DNA was cleaned using the Agencourt AMPure XP SPRI beads (Beckman Coulter, Australia), followed by end repair and A-tailing of the fragments using unique Illumina 9 nt p5 and p7 barcode adaptors (IDT × Gen Custom Universal Blocking Oligos) to generate dual-indexed libraries. Library preparation was conducted with KAPA Hyper Prep Kit (KAPA Biosystems, Wilmington, MA). We performed preliminary tests to compare different scales of KAPA sample library reactions for target capture efficiency. Half-scale KAPA sample library reactions were the best compromise in terms of target capture efficiency and coverage of target genes as the proportion of on-target reads for half-scale libraries was comparable to the full-scale libraries. Equimolar amounts of each amplified library were pooled in reactions of twelve indexed samples each (total of 500 ng DNA per 12-plex pool) using a Biomek FX^P^ laboratory automation workstation liquid handling system (Beckman Coulter, Australia). Hybridization of customised RNA baits with capture pools was performed at 65 °C for 24 hours. Dynabeads® MyOne™ Streptavidin C1 magnetic beads (Thermo Fisher Scientific, Australia) were used to isolate biotinylated DNA, and amplification of bead-bound enriched libraries (9-10 cycles) was performed using KAPA HiFi HotStart Ready Mix (KAPA Biosystems, Wilmington, MA, USA) and adapter-specific primers. Concentration of all enriched libraries was determined with a Qubit fluorometer (Thermo Fisher Scientific, Australia), and library size with Tapestation (Agilent, Santa Clara, CA, USA) for subsequent library titration and next-generation sequencing. Libraries were titrated with KAPA Library Quantification Kit (KAPA Biosystems, Wilmington, MA). These methods are expanded versions of descriptions in our related work (ref.^7^).

### Targeted resequencing, sequence alignment and data analysis

**Fig. 1:**
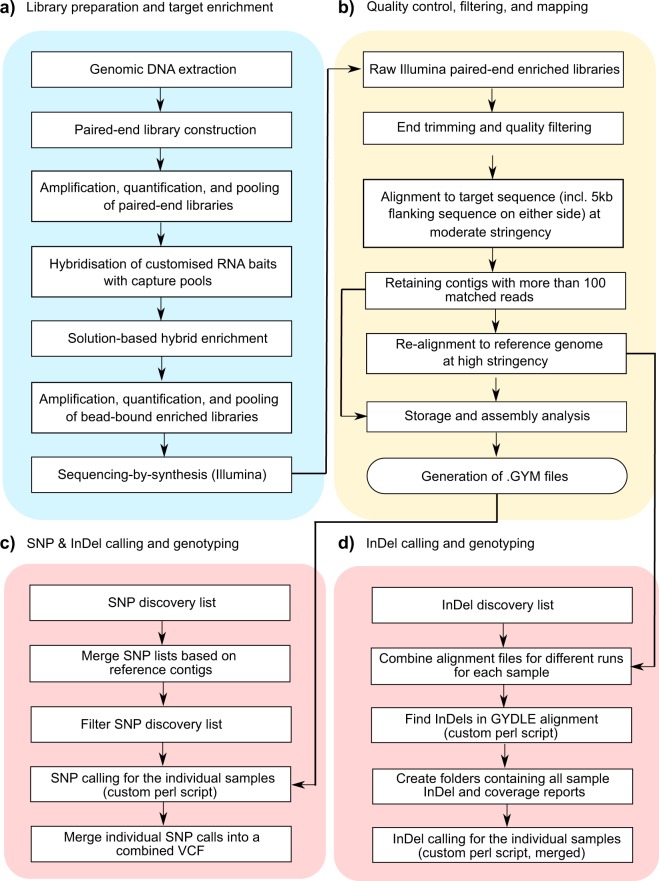
Schematic overview of the experimental and data analysis pipeline using GYDLE and custom algorithms. (**a**) Library preparation and target enrichment using MYbaits technology (MYcroarray®, Ann Arbour, MI, USA). (**b**) Quality control and filtering workflow using nuclear v3.2.6 (GYDLE Inc., Montreal, Canada). (**c**) SNP and (**d**) InDel calling and genotyping pipelines using a combination of nuclear v3.2.6, gym-build v2.6.16, coverage v2.6.16 and findsnp v2.6.16 (GYDLE) software and custom algorithms written in Perl.

Each pooled library was sequenced on an Illumina HiSeq3000 instrument using three lanes to generate about 0.5 million 2 × 150 bp paired-end reads per sample^9^. In addition to methods described in our related work (ref.^7^), here we include the discovery and genotyping procedures of InDels and a detailed and expanded description of the bioinformatics pipeline. A schematic overview of our data analysis pipeline for filtering, alignment, variant discovery, and genotype calling of target gene sequences is provided in Fig. 1.

Fastq sequence files were post-run filtered using nuclear software v3.6.16 (GYDLE Inc., Montreal, Canada) filtering sequences based on a minimum q-score of 20 and a minimum length of 50 bp. The resulting high-quality (HQ) reads were aligned to the target molecules, including 5 kb flanking regions on either side of the genes used in the RNA probe design, using nuclear software with low stringency high-scoring segment pairs (HSPs) with minimum read length 30 bp, with sensitivity 13 (consecutive identities), and up to 3 mismatches per window of 30 bases (~90% identity). Reads aligning to genomic regions targeted in the capture assay were retained as on-target reads. These on-target reads were re-aligned using nuclear software with higher stringency (HSPs with minimum read length 50 bp, sensitivity 25 and at most 3 mismatches per window of 50 bases) to the latest release of the barley reference genome assembly^4^ (IBSC v2). Genetic coordinates were converted to match the latest assembly^4^ (IBSC v2).

After quality filtering 895 barley genotypes with sufficient HQ reads were retained and used for alignment, variant discovery and genotype calling. The final dataset consisted of 1.12 billion 150-bp paired-end reads (203 Gb of raw data). The median filtered read number was 1 M reads per sample. Only 13% of the samples had less than 0.5 M reads while 8% of the samples had more than 2 M reads (see Table 1**)**.

**Table 1:** Sequencing and alignment values for total read number and alignment rate. The table lists the arithmetic mean, the minimal and maximal values as well as the standard deviation (Std dev) for the total set (first line) and the respective subsets of spring, winter/facultative, two-row, and six-row type barley.

	Total reads (raw)	Total reads (filtered)	Total reads (aligned)	Aligned reads (%)
**Mean**				
All	1,237,129	1,236,974	250,585	22.52
Two-row	1,241,038	1,240,944	250,895	22.50
Six-row	1,218,497	1,217,753	251,282	22.81
Winter/facultative	1,046,415	1,045,641	253,353	23.78
Spring	1,257,319	1,257,312	250,377	22.51
**Min**				
All	84,134	84,074	8,436	2.77
Two-row	84,134	84,074	8,436	2.77
Six-row	179,215	179,022	19,027	2.92
Winter	366,368	365,904	29,087	5.35
Spring	84,134	84,074	8,436	2.77
**Max**				
All	14,327,607	14,324,144	1,220,662	52.89
Two-row	14,327,607	14,324,144	1,220,662	52.89
Six-row	10,017,062	10,014,212	741,609	43.68
Winter/facultative	2,094,153	2,093,049	646,642	42.95
Spring	14,327,607	14,324,144	1,220,662	52.89
**Std dev**				
All	1,366,658	1,365,983	168,172	10.69
Two-row	1,385,747	1,385,044	168,421	10.59
Six-row	1,207,885	1,207,492	169,592	11.59
Winter/facultative	408,999	408,654	152,969	10.86
Spring	1,488,381	1,487,633	172,289	10.88

### Specificity and sensitivity

In total, the captured reads aligned to 193 distinct regions of the *Hordeum vulgare* assembly ASM32608v1 reference genome corresponding to all intended target genes. The average target sensitivity, interpreted as the percentage of target bases covered by sequence reads, was 88% (Table 2). Two target gene copies (DQ492699 and DQ492698) had insufficient coverage across samples but were retained in the target list. Targeted enrichment values (mean target coverage over the mean genome-wide coverage) for each sample were calculated from Illumina sequencing data using the formula from^12^ (see equation 1). The overall mean enrichment factor was 1,389.4.

1 $$Target\hspace{2.77626pt}enrichment\hspace{2.77626pt}factor=\frac{\frac{Number\hspace{2.77626pt}of\hspace{2.77626pt}reads\hspace{2.77626pt}that\hspace{2.77626pt}map\hspace{2.77626pt}to\hspace{2.77626pt}the\hspace{2.77626pt}target\hspace{2.77626pt}region}{Total\hspace{2.77626pt}number\hspace{2.77626pt}of\hspace{2.77626pt}reads}}{\frac{Target\hspace{2.77626pt}region\hspace{2.77626pt}size}{Haploid\hspace{2.77626pt}genome\hspace{2.77626pt}size}}$$

**Table 2:** Quality measures for the target enrichment for the total population. The table lists mean values for genome-wide and target coverage, the enrichment factor calculated from that, the normalized mean target coverage, the fractions of the genome and the target covered by any read and by at least 10 reads for the total barley set.

Quality measure	Total
Total number of reads (filtered)	21,236,974
Number of reads that map to target region	548,074
Target region size (bp)	589,819
Haploid genome size (bp) (*Hordeum vulgare* assembly ASM32608v1)	4,045,300,851
Mean genome-wide coverage	0.00015
Mean target coverage	0.2
Mean target length (bp)	3087.2
Enrichment factor	1,389.4
Mean fraction of target covered (%)	88

### Variant discovery and genotype calling

The capture assay was designed to capture 600 kb of target sequence which corresponded to 1.9 Mbp of target gene space in the current barley genome assembly^4^ (IBSC v2). SNP variant discovery as well as genotype calling was performed using custom Perl scripts to produce a VCF version 4.2 genotype file based on the alignment files (described in ref.^7^). To eliminate population-specific bias due to the composition of the SNP discovery panel, the entire population was selected to discover SNPs.

The SNP discovery pipeline identified a total of 467,339 SNPs, of which 29,822 were within gene-coding regions, including 6,030 SNPs which were captured within the 174 targeted phenology gene regions (VCF_SNP.vcf.gz^9^). On a per sample basis, about 75% of the SNP detected within the captured regions had a mean coverage depth of 25 reads per SNP. As expected for an inbreeding population, the rate of heterozygosity for each variety was very low (approximately 2% on average). Out of the 174 phenology genes, 168 genes had at least one SNP. A maximum of 445 SNPs were documented in *ELF7*, followed by *MADS25-3* and *MADS25-2* with 378 and 291 SNPs, respectively. Genes with most SNPs per kb were *GA3ox1* (51 SNP kbp^−1^), *CBF8A* (40 SNP kbp^−1^), and *CBF3* (35 SNP kbp^−1^). Barley varieties with most detected alternate alleles at SNP positions detected within the target capture region were OWB Parent Dom (1,754), Esperance Orge 289 (1,624), and Dicktoo (1,621), whereas MN Brite (92), Legacy (78), and Tradition (77) showed the least number of alternate alleles at SNP positions detected within the target capture region.

The InDel calling pipeline identified a total of 135,111 InDels with a size range of 1– 671 nucleotides, of which 8,400 were within gene-coding and 1,581 were within the targeted phenology gene regions (VCF_INDEL.vcf.gz^9^). On a per sample basis, about 80% of InDels detected within the captured regions had a mean coverage depth of 25 reads per InDel. The rate of heterozygosity for each variety was similarly low as for SNPs (approximately 3% on average). Out of the 174 phenology genes, 162 genes had at least one InDel. A maximum of 83 InDels were present in *ELF7*, followed by *MADS25-3* and *MADS25-2* with 65 and 57 InDels, respectively. Genes with most InDels per kb were *GA3ox1* (9.4 InDel kbp^−1^), *GA20ox3* (8.3 SNP kbp^−1^), and *CBF4A* (8.2 SNP kbp^−1^). Barley varieties with most detected alternate alleles at InDel positions detected within the target capture region were OWB Parent Dom (52), Smooth Awn 86 (52), and 91HBSN24 (50), whereas 38 varieties had no alternate alleles at any of the InDel positions detected within the target capture region, including several Australian (e.g. WI4560) and North American varieties (e.g. CDC Battleford, Chapais).

### Variant prediction

**Fig. 2:**
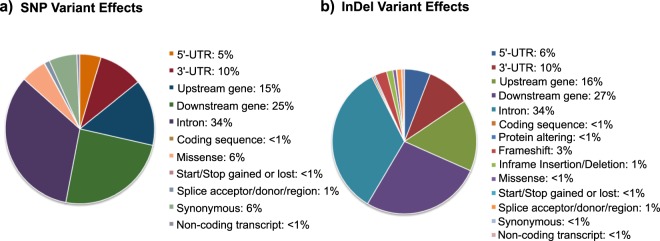
Consequences of genetic polymorphisms identified by targeted enrichment sequencing of flowering time genes categorized by Ensembl Variant Effect Predictor. A total of 6,030 SNP variants are categorized and percentages of potential consequences is given a) for all consequences. For InDels, a total of 1,581 variants are categorized and percentages of potential consequences is given c) for all consequences. See Ensembl Variant documentation for explanation of consequence categories (http://www.ensembl.org/info/genome/variation/predicted_data.html#consequences).

Using the target enrichment strategy, we identified regulatory (3′-UTR/5′-UTR/intron/upstream/downstream) as well as coding (frameshift/splice variant/missense/synonymous) novel SNP allelic variants for 170 out of the 174 targeted candidate genes regulating flowering time in barley. In total, 6,030 SNPs were detected within the target gene regions, and Variant effect prediction of all SNP captured within the target regions was performed using the Ensembl Variant Effect predictor toolset^13^ (Ensembl Variant Effect Predictor web interface http://www.ensembl.org/vep). Most SNPs were detected in the introns and downstream gene regions (Fig. 2a). Approximately 26% and 34% of all SNP effects fell within exonic and intronic sequences, respectively, based on the predictions of the Variant Effect predictor (VEP) tool^13^. Of the SNP variants discovered within the target gene regions, we found an impact (deleteriousness) distribution of approximately 7% (low), 87% (modifier), 5.5% (moderate), and 0.2% (high). Out of 174 targeted genes involved in flowering time, we found 132 genes with mutations having moderate effects, and 30 genes having large effects, including many known genes (such as *ELF3*, *PPD-H1*, *LFY1*, and *AGLG1*).

Approximately 22% and 34% of all InDel effects fell within exonic and intronic sequences, respectively. Similar as for the detected SNPs, about a third of all InDels were detected in intronic regions (Fig. 2b). For all InDel variants, we found an impact (deleteriousness) distribution of 0.5% (low), 94% (modifier), 2.5% (moderate), and 3% (high). Out of 174 targeted genes involved in flowering time, we found 53 genes with mutations having moderate effects, and 69 genes having large effects, including many known flowering time-related genes (such as *PHYC*, *FD*, *CO2*, and *ELF3*).

Thus, the assembled sequences and the extensive variation dataset obtained through targeted enrichment genome sequencing should support studies using forward and reverse genetic approaches.

## Data Records

Metadata for the germplasm collection and phenology gene targets is available in files Germplasm.xlsx, and Phenology_Genes.xlsx, respectively^9^. The SNP and InDel genotype matrices are available in VCF (Variant Call File format) (files VCF_SNP.vcf.gz, and VCF_INDEL.vcf.gz, respectively), and KASP primer sequences and KASP genotyping results are made available in files KASP_Primers.xlsx and KASP_Genotyping.xlsx, respectively (also^9^). The raw sequence data (.fastq files) are stored at ArrayExpress^8^.

## Technical Validation

### Genotypic assessment of biological samples

To support the technical quality of the dataset, sequences of InDel and SNPs from the targeted enrichment were utilized to develop and test genetic molecular markers (KASP_Primers.xlsx^9^. Uniplex Kompetitive Allele Specific PCR (KASP) technology was used to develop genetic markers, which offers a low-cost medium throughput genotyping platform suitable for fast and accurate screening of thousands of genotypes^14,15^. This makes KASP suitable for MAS in many breeding programs. Marker selection ensured that a vast range of allelic variation was captured for major phenology genes in barley inlcuding *PPD-H1*, *PhyC*, and *FT1*. Genomic DNA of Australian barley cultivars Vlamingh, Buloke, Commander and Fleet, as well as 60 other barley varieties from the phenology set were extracted as described previously.

For KASP assays developed in this study, complete coding sequences were obtained from the latest version of the barley reference genome assembly^4^ (IBSC v2). Sequences of 64 SNPs (described in ref.^7^) and 3 InDels present in candidate genes were converted into 67 Kompetitive Allele Specific PCR (KASP) assays to detect the specific parental allele for MAS in barley breeding. Allele-specific and common primers for each KASP marker were designed using Geneious® software version 10.2.3 (Biomatters Ltd., NZ) following standard KASP guidelines. The allele-specific primers (AS1 and AS2) were designed carrying the standard FAM (5′-GAAGGTGACCAAGTTCATGCT-3′) and VIC (5′-GAAGGTCGGAGTCAACGGATT-3′) tails, and with the targeted SNP or Indel at the 3′ end. A common primer (C) was designed so that the total amplicon length was between 100 and 150 bp. Assays were tested in 384-well formats and set up as ~5 µl reactions (15 ng/µl dry DNA, 2.5 µl of 1 × KASP master mixture, and 0.1 µl of primer mixture). The primer mixture comprised of a final concentration of 30 µM of the common primer, and 10 µM of each tailed primer. PCR cycling was performed using the following protocol: hot start at 94 °C for 15 min, followed by ten touchdown cycles (94 °C for 20 s; touchdown at an initial temperature of 61 °C and decreasing by −0.6 °C per cycle for 60 s) achieving a final annealing temperature of 55 °C, followed by 26 additional cycles of annealing (94 °C for 20 s; 55 °C for 60 s). Fluorescence readings were performed at 37 °C. Amplification was carried out using the ABI ViiA7 instrument (Applied Biosystems, Foster City, CA, USA), and plants were genotyped using KASP markers according to the manufacturer’s instructions (LGC Genomics, Hoddeson, UK). End-point genotyping was done using the ABI QuantStudio™ Real-Time PCR software v1.3.

In total, we designed and tested 67 KASP assays that distinguished between alleles for 31 key phenology genes. KASP markers were subsequently validated using subsets of the barley variety panel (6–47 varieties tested and confirmed per marker), including Australian cultivars Hindmarsh, La Trobe, Baudin, Scope, Commander, and Fleet. Individual genotyping using the KASP markers for barley accessions with contrasting alleles at the locus (allele 1 corresponds to the reference allele, allele 2 to the alternative allele, allele 0 to a heterozygous locus, and “.” to a missing data point) showed an agreement (“Y”) of 80–100% between the estimated allele from the target enrichment and sequencing (column “Resequencing”) and absolute values based on the KASP genotyping (column “KASP”, KASP_Genotyping.xlsx^9^). On average, more than 90% of all alleles were scored identically between the re-sequenced data and KASP genotyping.

## Usage Notes

The plant material described in this paper is publically available and can be made accessible under a Standard Material Transfer Agreement (SMTA).

## ISA-Tab metadata file

Download metadata file

## Data Availability

Custom codes are available from the corresponding authors on reasonable request.
